# Supplementary material for: Empirical methods for controlling false positives and estimating confidence in ChIP-Seq peaks
Source: BMC Bioinformatics. 2008 Dec 5;9:523. doi: 10.1186/1471-2105-9-523 (PMC2628906; doi:10.1186/1471-2105-9-523)
Supplement: Additional File 1 — A variety of html documents from the USeq web site detailing the available applications, their best usage, output file type descriptions, command line menus, etc. [file 1471-2105-9-523-S1.zip › USeqUserGuides/JavaDoc.zip!/JavaDoc/bioroot/plasmid/PlasmidModify.html]

PlasmidModify


|  |  |  |
| --- | --- | --- |
| **Overview** \| **Package** \| **Class** \| **Use** \| **Tree** \| **Deprecated** \| **Index** \| **Help** | |  |
| **PREV CLASS**   **NEXT CLASS** | **FRAMES**    **NO FRAMES**     **All Classes** |  |
| SUMMARY: NESTED \| FIELD \| CONSTR \| METHOD | DETAIL: FIELD \| CONSTR \| METHOD |  |


---


## bioroot.plasmid Class PlasmidModify

```
java.lang.Object
  javax.servlet.GenericServlet
      javax.servlet.http.HttpServlet
          bioroot.plasmid.PlasmidModify
```

**All Implemented Interfaces:**: java.io.Serializable, javax.servlet.Servlet, javax.servlet.ServletConfig

---

``` public class PlasmidModify extends javax.servlet.http.HttpServlet ```

Servlet to modify plasmids using PlasmidBeans referenced from a Session object.

**Author:**
:   nix

**See Also:**: Serialized Form

---

**Constructor Summary**

`PlasmidModify()`


| **Method Summary** | |
| --- | --- |
| `static java.lang.String` | `deletePlasmid(PlasmidBean plasmidBean, UserBean userBean, DBUtil bioRoot)`             Attempts to delete an plasmid, if sucessful, returns a "" String, otherwise an error message. |
| `void` | `doGet(javax.servlet.http.HttpServletRequest request, javax.servlet.http.HttpServletResponse response)` |
| `void` | `doPost(javax.servlet.http.HttpServletRequest request, javax.servlet.http.HttpServletResponse response)` |
| `static boolean` | `subtractAndSetPlasmidBeans(PlasmidBean[] plasmidBeans, javax.servlet.http.HttpSession session)`             Subtracts a bean from the PlasmidBean[] and resets it in plasmidBeansToEdit |
| `static java.lang.String` | `updatePlasmid(PlasmidBean bean, UserBean userBean, java.util.HashMap nameValue, javax.servlet.http.HttpServletRequest request, DBUtil bioRoot)`             Attempts to update a PlasmidBean in the database, only checks that the name is unique if it has changed. |

**Methods inherited from class javax.servlet.http.HttpServlet**

`service`

**Methods inherited from class javax.servlet.GenericServlet**

`destroy, getInitParameter, getInitParameterNames, getServletConfig, getServletContext, getServletInfo, getServletName, init, init, log, log`

**Methods inherited from class java.lang.Object**

`equals, getClass, hashCode, notify, notifyAll, toString, wait, wait, wait`

**Constructor Detail**

### PlasmidModify

```
public PlasmidModify()
```


**Method Detail**

### doGet

```
public void doGet(javax.servlet.http.HttpServletRequest request,
                  javax.servlet.http.HttpServletResponse response)
           throws javax.servlet.ServletException,
                  java.io.IOException
```

:   **Overrides:**: `doGet` in class `javax.servlet.http.HttpServlet`
:   **Throws:**: `javax.servlet.ServletException`: `java.io.IOException`

---


### doPost

```
public void doPost(javax.servlet.http.HttpServletRequest request,
                   javax.servlet.http.HttpServletResponse response)
            throws javax.servlet.ServletException,
                   java.io.IOException
```

:   **Overrides:**: `doPost` in class `javax.servlet.http.HttpServlet`
:   **Throws:**: `javax.servlet.ServletException`: `java.io.IOException`

---


### deletePlasmid

```
public static java.lang.String deletePlasmid(PlasmidBean plasmidBean,
                                             UserBean userBean,
                                             DBUtil bioRoot)
```

:   Attempts to delete an plasmid, if sucessful, returns a "" String, otherwise an error message.

---


### updatePlasmid

```
public static java.lang.String updatePlasmid(PlasmidBean bean,
                                             UserBean userBean,
                                             java.util.HashMap nameValue,
                                             javax.servlet.http.HttpServletRequest request,
                                             DBUtil bioRoot)
```

:   Attempts to update a PlasmidBean in the database, only checks that the name is unique if it has changed.
    If the update is sucessful, the message will be "", otherwise an error message will be returned.

---


### subtractAndSetPlasmidBeans

```
public static boolean subtractAndSetPlasmidBeans(PlasmidBean[] plasmidBeans,
                                                 javax.servlet.http.HttpSession session)
```

:   Subtracts a bean from the PlasmidBean[] and resets it in plasmidBeansToEdit


---


|  |  |  |
| --- | --- | --- |
| **Overview** \| **Package** \| **Class** \| **Use** \| **Tree** \| **Deprecated** \| **Index** \| **Help** | |  |
| **PREV CLASS**   **NEXT CLASS** | **FRAMES**    **NO FRAMES**     **All Classes** |  |
| SUMMARY: NESTED \| FIELD \| CONSTR \| METHOD | DETAIL: FIELD \| CONSTR \| METHOD |  |


---
